# Investigating the effects of percutaneous endovascular aneurysm repair for abdominal aortic aneurysm on the lumen size of the common femoral artery

**DOI:** 10.1186/s42155-024-00476-0

**Published:** 2024-09-10

**Authors:** Wilson Wei Xiang Ong, Hsien Ts’ung Tay, Tze Tec Chong

**Affiliations:** 1 Duke-NUS Medical School, Singapore, Singapore; 2 Department of Vascular Surgery, Singapore General Hospital, Singapore, Singapore

**Keywords:** Abdominal aortic aneurysm, Percutaneous endovascular aneurysm repair, Common femoral artery, Inner diameter, Outer diameter, Surgical outcomes

## Abstract

**Background:**

Percutaneous endovascular aneurysm repair (PEVAR) is the definitive therapy of choice for abdominal aortic aneurysms worldwide. However, current literature regarding the anatomic changes in the common femoral artery (CFA) post-PEVAR is sparse and contradictory, and a significant proportion of these studies did not control for the potential confounding effects of ethnicity. Thus, this study aims to investigate the anatomical effects of PEVAR on the CFA using an Asian study cohort.

**Methods:**

Between January 2019 and September 2023, the records of 113 patients who received PEVAR were reviewed. Groins with previous surgical interventions were excluded. The most proximate pre- and postoperative CT angiography of patients receiving PEVAR via the Perclose ProGlide™ Suture-Mediated Closure System were retrospectively analysed for changes in both the CFA inner luminal diameter (ID) and outer diameter (OD), the latter also encompassing the arterial walls. Access site complications within 3 months post-PEVAR were also recorded per patient.

**Results:**

One hundred seventeen groins from 60 patients were included in this study, with 1 report of pseudoaneurysm. The CFA ID exhibited a 0.167 mm decrease (*p*-value = 0.0403), while the OD decreased by 0.247 mm (*p*-value = 0.0107). This trend persisted when the data was separately analysed with the common cardiovascular risk factors of diabetes mellitus, hypertension and hyperlipidaemia.

**Conclusion:**

Our analysis demonstrated a statistically significant decrease in the CFA diameters post-PEVAR. However, the percentage changes were below established flow-limiting values, as reflected by the single access site complication reported. Hence, our findings give confidence in the safety profile of this procedure, even with the reported smaller baseline CFA lumen size in Asians. Moving forward, similar longer-term studies should be considered to characterise any late postoperative effects.

**Graphical Abstract:**

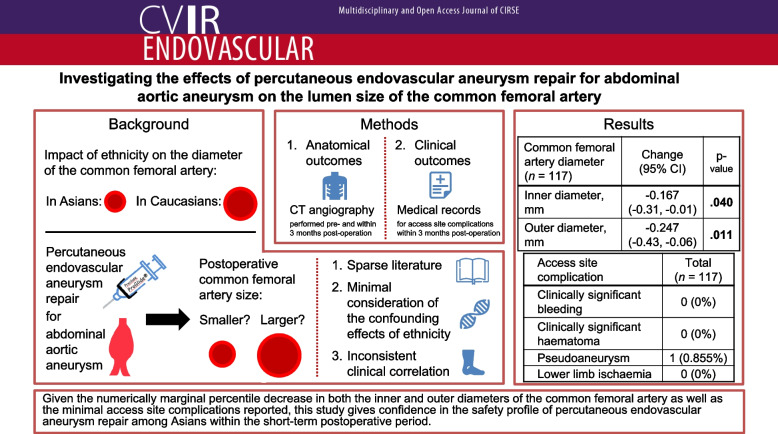

## Introduction

Over the past two decades, endovascular aneurysm repair has consistently demonstrated a favorable morbidity and recovery profile vis-à-vis open aneurysm resection [1–3]. Further improvements in the perioperative course, including reductions in operating time and length of stay, were facilitated by the introduction of percutaneous access (PEVAR), thereby solidifying the current role of PEVAR as the mainstay of AAA management [3–7]. One such platform is the widely utilised Preclose ProGlide™ system (Abbott Vascular, Green Oaks, Illinois, USA), a suture-mediated closure device that boasts an established safety and efficacy record even against its popular counterparts [7–9].

However, the utilisation of PEVAR does not guarantee the complete elimination of adverse perioperative outcomes [9–11]. This includes access-related groin complications, which the characteristically minimally invasive nature of PEVAR most directly addresses [12–14]. Previous studies have attributed this observation to the unchanged rates of pseudoaneurysms and potentially flow-limiting arterial thrombosis, both widely recognised as being among the more common iatrogenic vascular sequelae [10, 14, 15].

The effects of PEVAR on the diameter of the common femoral artery (CFA), the default PEVAR access site [13], have seen cyclic surges of interest from various groups as a direct indicator of procedural safety and, on a theoretical level, an anatomical basis for vascular access site sequelae. However, existing literature has been contradictory, albeit with more studies reporting no significant differences post-PEVAR [11, 16–20]. Additionally, attempts in correlating the anatomic findings to resultant acess site complications are inconsistent, and the number of studies that actively considered the potential confounding influence of ethnicity remains undesirably sparse, despite the recent finding of a smaller baseline CFA inner diameter (ID) among Asians compared to Caucasians [21].

Hence, this study aims to investigate the anatomical effects of PEVAR on an Asian population by measuring the resultant changes in both the CFA ID as well as the outer diameter (OD), the latter of which also includes the outer luminal wall [20]. The timeframe of this study will be limited to the short-term 3-month postoperative period, given the reported highest reintervention rates within this window compared to other timepoints post-PEVAR [22].

## Materials and methods

### Patient selection

This is an Institutional Review Board-approved study with waiver of informed consent. A retrospective cohort study was conducted using data from patients who received PEVAR for AAA between January 2019 and September 2023 at Singapore General Hospital. Patients who received PEVAR via a closure device other than the Preclose ProGlide™ system as well as those with an incomplete computed tomography (CT) angiography series were excluded. The latter exclusion criteria also included patients who received their first postoperative CT angiography more than 90 days post-PEVAR. Subsequent sample selection was conducted via a per-groin approach, with any prior history of groin interventions or access (including previous endarterectomies) as well as intraoperative conversion to femoral cutdown serving as the two exclusion criteria.

Demographic information of the final study cohort was extracted from our institution’s Electronic Medical Records and anonymised. The specific fields of interest were age, gender, active antiplatelet therapy, active anticoagulation therapy as well as the common comorbid cardiovascular risk factors of body mass index, smoking status, diabetes mellitus (DM), hypertension (HTN), hyperlipidaemia (HLD), ischaemic heart disease and peripheral arterial disease (PAD).

Groin complications were extracted from clinical notes in our institution’s Electronic Medical Records within 3 months post-PEVAR before being subjected to patient deidentification. Specifically, the complications of interest were clinically significant bleeding [23], clinically significant haematoma [24], pseudoaneurysm as well as lower limb ischaemia.

### Preclose technique

Standard PEVAR via the preclose technique was performed for all patients. This involved an ultrasound-guided retrograde puncture of the CFA that was followed by the sequential deployment of Preclose ProGlide™ devices and the necessary sheaths, all of which were between 12 and 20F in calibre. Access site closure was then mediated via the extracorporeal sutures. A detailed description of PEVAR via the preclose technique has been described in previous studies [25–27].

### Acquisition of the anatomical details of the CFA

**Fig. 1:**
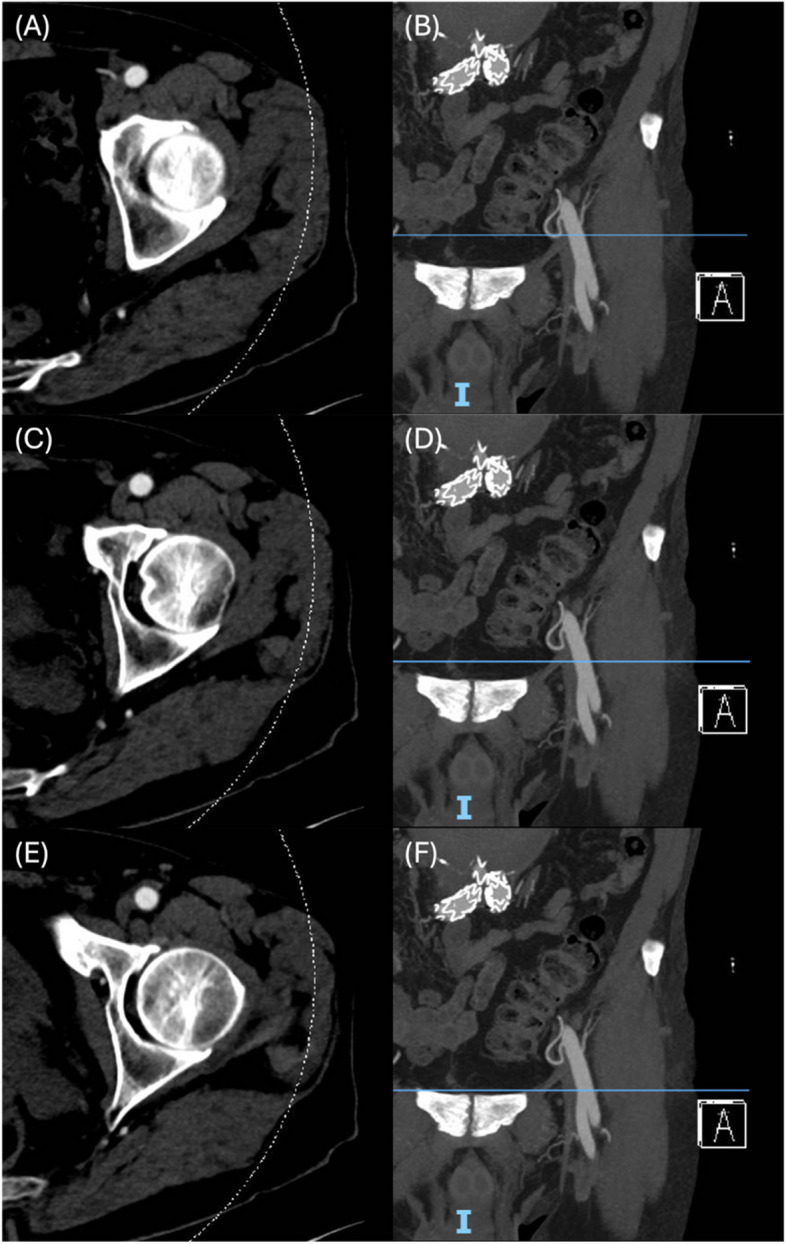
Postoperative computed tomography series of a left common femoral artery illustrating the anatomical locations at which the diameters of the common femoral artery were recorded.** A**, **B** Respective axial and coronal images of the common femoral artery at the proximal origin below the inguinal ligament. **C**, **D** Respective axial and coronal images at the midpoint of the common femoral artery. **E**, **F** Respective axial and coronal images of the distal common femoral artery just proximal to the profunda bifurcation

CT scans obtained at the first postoperative visits were compared against the last available preoperative CT scans. Both scans were performed according to standard department protocols using either the Siemens SOMATOM Definition Flash (Siemens Healthineers AG, Forchheim, Bavaria, Germany) or the Canon Aquilion Prime (Canon Medical Systems Corporation, Otawara-shi, Tochigi, Japan) at 3 mm slice thickness. The CT angiography protocol involved injection of 70 to 80 ml of Omnipaque 350 (GE Healthcare, Milwaukee, Wisconsin, USA) at 4 ml/s followed by a 30 ml saline washout at 4 ml/s. Quantitative assessment of the CFA was then performed on a per-groin basis by measuring the CFA ID and OD on Vue Motion (Carestream Health Inc, Rochester, New York, USA) with electronic calipers. This was performed using the abdomen window with a window level of 400 and window width of 50. To best capture the anatomical effects along the entire CFA, measurements were recorded at three discrete sites: 1) at the proximal CFA below the arterial origin at the inguinal ligament, 2) at the distal CFA immediately above the profunda bifurcation, and 3) along the central CFA tract, measured as the midpoint between points 1) and 2). The proximal and distal points of interest were correlated on the coronal images before the measurements were obtained from the axial images (Fig. 1). This was performed by a single medical student under the guidance of an experienced senior vascular surgical consultant.

### Statistical analysis

Measurements of the CFA diameters at the three anatomic locations were averaged per groin and checked for normality via the Shapiro–Wilk test. Subsequent analysis was conducted accordingly via paired *t-*tests and paired Wilcoxon signed-rank tests for datasets following normal and non-normal distributions respectively. All statistical analyses were performed with RStudio version 2023.12.1.402 (RStudio, Boston, Massachusetts, USA).

## Results

### Patient population

**Fig. 2:**
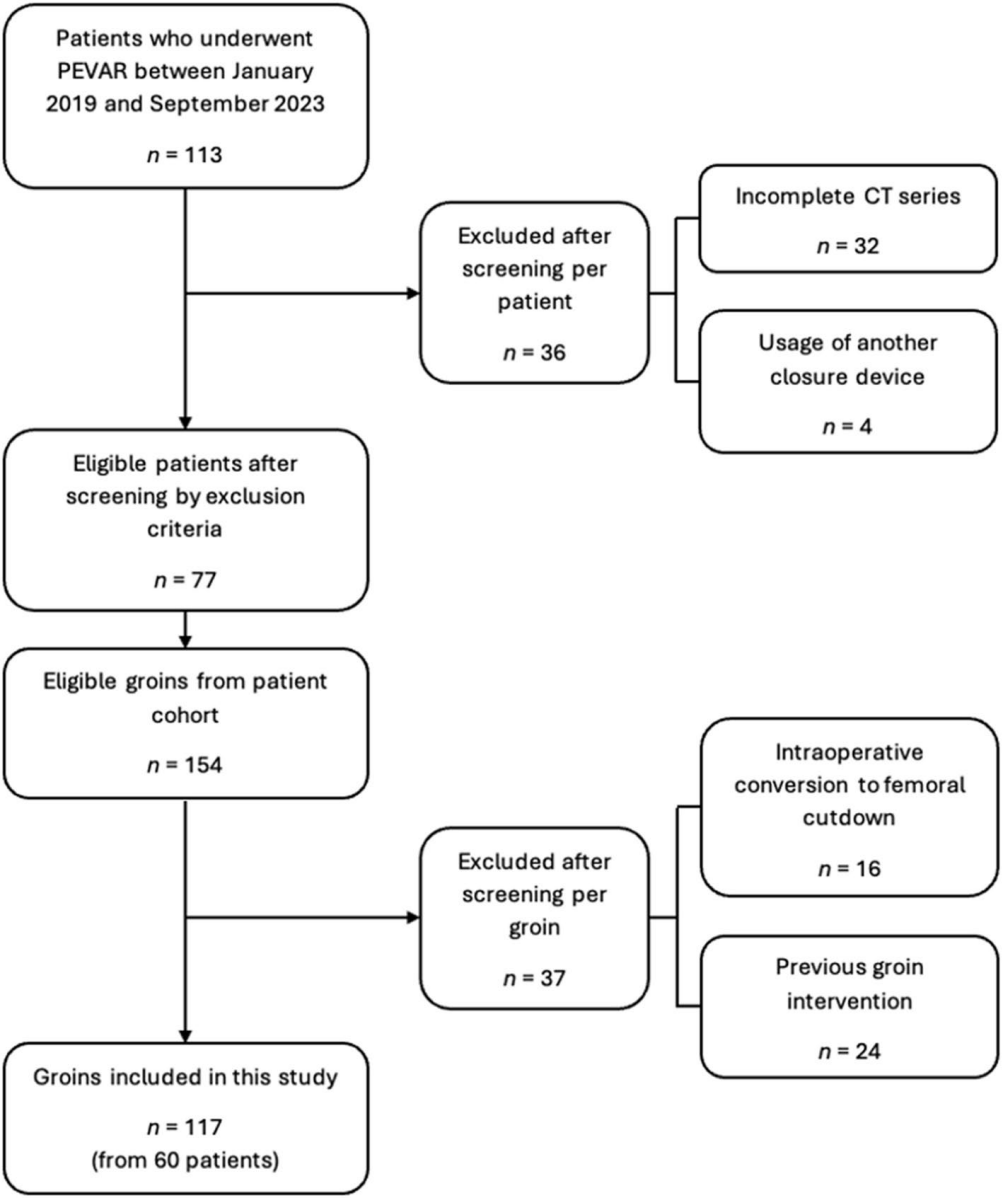
Flowchart illustrating the study cohort selection process

Between January 2019 and September 2023, a total of 113 patients received PEVAR for AAA at our institution. Of these patients, 36 were excluded, with the vast majority being due to incomplete CT series. Subsequent per-groin analysis resulted in the exclusion of 37 groins. As such, a total of 117 groins from 60 patients were included in this study. Figure 2 depicts the study cohort selection process, while the demographic characteristics of these patients are summarised in Table 1. On average, the postoperative CT scans were obtained at 39.2 ± 24.9 days post-PEVAR, while the preoperative CT scans were obtained at 52.0 ± 45.2 days pre-PEVAR. The single case of pseudoaneurysm constitutes the sole early access-site complication reported (Table 2).

**Table 1:** Baseline characteristics of the study population

Variable	Total (*n* = 60)
Gender	
Male	53 (88.3%)
Female	7 (11.7%)
Age, years	73.1 ± 8.54
Body mass index, kg/m^2^	24.5 ± 4.13
Smoking status	
Active smoker	17 (28.3%)
Ex-smoker	20 (33.3%)
Non smoker	23 (38.3%)
Diabetes mellitus	9 (15.0%)
Hypertension	46 (76.7%)
Hyperlipidaemia	48 (80.0%)
Ischaemic heart disease	28 (48.7%)
Peripheral arterial disease	2 (3.33%)
Antiplatelet therapy	
Aspirin	29 (48.3%)
Clopidogrel	1 (1.67%)
Dipyridamole	1 (1.67%)
Ticagrelor	3 (5.00%)
Anticoagulant therapy	
Apixaban	2 (3.33%)
Rivaroxaban	3 (5.00%)

Data presented as *n* (%) or mean ± standard deviation

**Table 2:** Reported early access site complications

Access site complication	Total (*n* = 117)
Clinically significant bleeding [23]	0 (0%)
Clinically significant haematoma [24]	0 (0%)
Pseudoaneurysm	1 (0.855%)
Lower limb ischaemia	0 (0%)

Data presented as *n* (%)

### Changes in CFA diameters

Table 3 summarises the changes in both the CFA ID and OD at a cohort level. As the ID data was shown to follow a non-normal distribution (Table 4), this dataset was analysed via paired Wilcoxon sign-rank tests while the normally distributed OD data was analysed with paired t-tests. Our analysis revealed a statistically significant decrease when comparing the pre- and postoperative IDs (9.31 ± 1.73 mm and 9.18 ± 1.84 mm respectively, p-value = 0.0403) as well as ODs (11.03 ± 1.99 mm and 10.79 ± 1.99 mm respectively, p-value = 0.0107). Additionally, changes in both the CFA ID and OD were separately analysed with the specific cardiovascular risk factors (Tables 5 and 6 respectively). Of these, it was noted that the presence of DM and HLD were separately associated with statistically significant decreases in both the CFA ID and OD, while the presence of HTN and ischaemic heart disease were only associated with a statistically significant decrease in the CFA OD. Conversely, the absence of PAD was associated with statistically significant decreases in both the CFA ID and OD, as is seen among aspirin-naïve patients.

**Table 3:** Comparison of the pre- and postoperative common femoral artery diameters

Common femoral artery diameter (*n* = 117)	Preoperative values	Postoperative values	Change	p-value
Inner diameter, mm	9.31 ± 1.73	9.18 ± 1.84	-0.167 (-0.31, -0.01)	**.040**
Outer diameter, mm	11.0 ± 1.99	10.8 ± 1.99	-0.247 (-0.43, -0.06)	**.011**

Data presented as mean ± standard deviation or mean (95% confidence interval). Boldface p-values indicate statistical significance (*p*-value < .05)

**Table 4:** Normality analysis of the change in the common femoral artery diameters post-PEVAR

Common femoral artery diameter	W statistic	p-value
Inner diameter	.97	**.015**
Outer diameter	.99	.46

Boldface p-values indicate statistical significance (*p*-value < .05)

**Table 5:** Subgroup analysis of the pre- and postoperative changes in the inner diameter of the common femoral artery

Variable	Preoperative CFA ID values	Postoperative CFA ID values	Change	p-value
Diabetes mellitus				
With (*n* = 18)	8.57 ± 1.59	8.18 ± 1.44	-0.428 (-0.71, -0.07)	**.016**
Without (*n* = 99)	9.44 ± 1.73	9.36 ± 1.85	-0.113 (-0.28, 0.06)	.21
Hypertension				
With (*n* = 89)	9.35 ± 1.76	8.24 ± 1.82	-0.160 (-0.33, 0.03)	.099
Without (*n* = 28)	9.18 ± 1.67	9.01 ± 1.88	0.223 (-0.16, 0.52)	.35
Hyperlipidaemia				
With (*n* = 94)	9.35 ± 1.80	9.18 ± 1.90	-0.203 (-0.38, 0.03)	**.027**
Without (*n* = 23)	9.13 ± 1.44	9.17 ± 1.58	0.00417 (-0.31, 0.33)	.99
Ischaemic heart disease				
With (*n* = 55)	8.97 ± 1.53	8.85 ± 1.61	-0.106 (-0.41, -0.11)	.25
Without (*n* = 60)	9.60 ± 1.88	9.48 ± 2.02	-0.166 (-0.35, 0.02)	.086
Peripheral arterial disease				
Without (*n* = 113)	9.37 ± 1.73	9.23 ± 1.85	-0.178 (-0.33, -0.02)	**.031**
Aspirin				
With (*n* = 56)	8.87 ± 1.39	8.83 ± 1.50	-0.107 (-0.35, 0.15)	.39
Without (*n* = 61)	9.70 ± 1.92	9.51 ± 2.06	-0.203 (-0.41, -0.01)	**.042**

Data presented as mean ± standard deviation or mean (95% confidence interval)

*CFA* Common femoral artery, *ID* Inner diameter

Boldface *p*-values indicate statistical significance (*p*-value < .05)

**Table 6:** Subgroup analysis of the pre- and postoperative changes in the outer diameter of the common femoral artery

Variable	Preoperative CFA OD values	Postoperative CFA OD values	Change (95% CI)	p-value
Diabetes mellitus				
With (*n* = 18)	10.6 ± 1.62	9.91 ± 1.68	-0.703 (-1.32, -0.09)	**.027**
Without (*n* = 99)	11.1 ± 2.04	11.0 ± 2.01	-0.703 (-0.36, 0.03)	.097
Hypertension				
With (*n* = 89)	11.1 ± 2.05	10.8 ± 2.01	-0.268 (-0.49, -0.04)	**.020**
Without (*n* = 28)	10.8 ± 1.77	10.6 ± 1.95	-0.178 (-0.53, 0.19)	.32
Hyperlipidaemia				
With (*n* = 94)	11.0 ± 2.11	10.8 ± 2.04	-0.260 (-0.46, -0.06)	**.011**
Without (*n* = 23)	11.0 ± 1.41	10.8 ± 1.81	-0.194 (-0.73, 0.35)	.47
Ischaemic heart disease				
With (*n* = 55)	10.7 ± 1.61	10.3 ± 1.57	-0.329 (-0.63, -0.03)	**.030**
Without (*n* = 60)	11.4 ± 2.26	11.2 ± 2.28	0.159 (-0.41, 0.09)	.21
Peripheral arterial disease				
Without (*n* = 113)	11.1 ± 1.98	10.9 ± 2.00	-0.262 (-0.46, -0.07)	**.009**
Aspirin				
With (*n* = 56)	10.6 ± 1.55	10.4 ± 1.50	-0.180 (-0.50, 0.14)	.26
Without (*n* = 61)	11.43 ± 2.25	11.1 ± 2.32	-0.307 (-005, -0.09)	**.007**

Data presented as mean ± standard deviation or mean (95% confidence interval)

*CFA* Common femoral artery, *OD* Outer diameter

Boldface *p*-values indicate statistical significance (*p*-value < .05)

## Discussion

Measuring the anatomical effects of PEVAR on the CFA provides a potential reference point to understand the factors governing adverse vascular outcomes post-PEVAR. However, existing literature has been sparse and contradictory. Hence, our study provides an analysis of the short-term anatomical effects of PEVAR on the CFA, specifically from an Asian study cohort.

Our analysis revealed a statistically significant decrease in the CFA ID post-PEVAR. Notably, this finding diverges from current literature, with all but one group reporting no significant difference (Dwivedi et al. is the sole exception with their study reporting a postoperative increase in the CFA ID) [16–20, 28]. Additionally, only three groups, namely Dwivedi et al., Lin et al. and Oğuzkurt et al., utilised CT angiography readings taken within the short-term postoperative period of 3 months post-PEVAR [17, 20, 28, 29], while only two groups, namely Ong et al. and Lin et al., controlled for ethnicity, which is a potential confounder given the reported smaller baseline CFA ID among Asians [19, 20, 21]. Regardless of the diversity in study cohorts, the reported postoperative access site complication rates did not surpass the current literature value of less than 3% [9, 10, 30]. With the postoperative access site complication rate in this study being congruent with these values, it may thus be suggested that our finding of a statistically significant decrease of 1.79% in the CFA ID did not translate into meaningful clinical impact on vascular health. Indeed, the ID is typically considered to have attained flow-limiting reduction upon reaching the literature threshold of 50%, while current definitions of interventional success allows for a remaining post-procedural ID reduction of up to 30% of the baseline [31, 32]. Unfortunately, the aforementioned studies utilising Asian study cohorts did not investigate postoperative complications [19, 20], and a longer study duration would be better positioned to illustrate the possible disconnect between statistical and clinical significance.

Additionally, it should be noted that the average preoperative CFA ID value from our Asian study cohort is instead more congruent with the literature trend among Caucasians [21, 33, 34]. While the classification of an “Asian” ethnicity necessarily implies significant inherent heterogeneity, our reported value also varies from the findings of previous studies that utilised data from within the same population, albeit spaced approximately a decade apart [19, 35, 36]. Given the complex multiethnic demography of Singapore, this discrepancy might have stemmed from a differential sampling of the various ethnicities (including the various subgroups of East Asians, South Asians and Southeast Asians, among others) in different proportions. Unfortunately, a subgroup ethnicity analysis was beyond the scope of this study. Moving forward, an observational study should be considered to investigate the average CFA ID value among Asians in the local setting, with special emphasis on subgroup analyses to control for Singapore’s multiethnic population.

Our findings regarding the statistically significant postoperative decrease in the CFA OD is also unexpected. As discussed by Lin et al., the only other group who also performed a similar investigation, recovery from PEVAR-induced iatrogenic arterial wall trauma should dominate the postoperative process, which would be reflected by a statistically significant increase in the CFA OD during the short-term perioperative period [20, 37, 38]. Indeed, this discrepancy in study outcomes could have stemmed from a difference in the data acquisition approach. Lin et al. acquired the CFA OD directly at the arterial puncture site, thus ensuring a theoretically accurate snapshot of the local inflammatory reaction secondary to the arterial puncture [20]. Conversely, our study methodology was designed to capture the change in the OD across the entire CFA. Thus, the results of both studies can be integrated together to suggest the safety of the PEVAR approach from a structural perspective, as the associated vascular injury is indeed highly localised and does not disrupt the overall property of the arterial wall, thereby allowing it to parallel the anatomic changes of the CFA ID.

Unlike previous studies, our analysis also investigated the impact of known cardiovascular risk factors on the changes in the CFA diameters post-PEVAR. Compared to the cohort-level analysis, our findings of an association between the various cardiovascular risk factors (namely DM, HTN and HLD) and a statistically significant decrease in CFA diameters is not unexpected. Indeed, they are a likely representation of the endothelial dysfunction and vasodilatory failure inherent in these conditions [39–42], thus reflecting the more prolonged recovery time required compared to their disease-free counterparts. Comparatively, the statistical significance among patients without PAD is likely a function of the other risk factors, given the inclusion of nearly the entire study cohort within this group. Moving forward, future studies investigating medium and long term effects of PEVAR on CFA diameters should also include a similar analysis to investigate the potential association between these risk factors and subsequent vascular sequelae.

However, the association between the absence of aspirin usage and a statistically significant decrease in CFA diameters is noteworthy. When juxtaposed against the contrary finding in patients with aspirin usage, this observation appears to suggest a protective anatomical effect of aspirin on the CFA. The molecular basis behind the anti-inflammatory and antiplatelet effects of aspirin has been extensively elucidated, and aspirin is still widely utilised for the prevention of cardiovascular disease progression and cerebrovascular events, among other vascular indications [43, 44]. However, patients typically undergo a washout period prior to PEVAR to manage the associated antiplatelet effects. Thus, this finding suggests a persistence of the anatomically beneficial effects of aspirin despite this washout period, although the absence of any clinically significant bleeding or haematoma reflects effective peri- and postoperative control of its antiplatelet effects. Indeed, a longer-term analysis focusing on postoperative outcomes would be better positioned to ascertain and discuss the potential clinical implications of this observation.

This study has several limitations. In addition to the aforementioned short-term focus of this study, the single-centre approach and retrospective nature of this study may have resulted in some inherent observational and selection bias. The exclusion of 32 patients from the initial cohort of 113 for incomplete CT angiography may also serve as an additional source of bias. Lastly, axial projections obtained during CT angiography do not always present a true perpendicular view of the CFA. However, this may be compensated by the comparative nature of the paired statistical tests utilised in this study.

## Conclusion

This study demonstrated a statistically significant decrease in CFA diameters among an Asian study cohort during the short-term postoperative period post-PEVAR, with the cardiovascular risk factors of DM, HTN and HLD serving as individual contributing factors. However, the numerically marginal decrease, coupled with the minimal number of short-term access site complications reported, provides strong support for the safety profile of PEVAR. Moving forward, further studies should be considered to evaluate the persistence of our observations into the later postoperative periods.

## Data Availability

The datasets used and/or analysed during the current study are regulated by the local Personal Data Protection Act 2012 and are thus not publicly available.

## Abbreviations

AAA: Abdominal aortic aneurysm
CFA: Common femoral artery
CT: Computed tomography
DM: Diabetes mellitus
ID: Inner diameter
HLD: Hyperlipidaemia
HTN: Hypertension
PAD: Peripheral arterial disease
PEVAR: Percutaneous endovascular aneurysm repair
OD: Outer diameter
